# *De novo *sequencing and characterization of *Picrorhiza kurrooa *transcriptome at two temperatures showed major transcriptome adjustments

**DOI:** 10.1186/1471-2164-13-126

**Published:** 2012-03-31

**Authors:** Parul Gahlan, Heikham Russiachand Singh, Ravi Shankar, Niharika Sharma, Anita Kumari, Vandna Chawla, Paramvir Singh Ahuja, Sanjay Kumar

**Affiliations:** 1 Biotechnology Division, CSIR-Institute of Himalayan Bioresource Technology (Council of Scientific and Industrial Research), P.O. Box No. 6, Palampur 176 061, Himachal Pradesh, India; 2 Studio of Computational Biology & Bioinformatics, CSIR-Institute of Himalayan Bioresource Technology (Council of Scientific and Industrial Research), P.O. Box No. 6, Palampur 176 061, Himachal Pradesh, India

**Keywords:** Mevalonate, Gene expression, 2-C-methyl-D-erythritol 4-phosphate, Next generation sequencing, Phenylpropanoid, *Picrorhiza kurrooa*, Picrosides, Transcriptome

## Abstract

**Background:**

*Picrorhiza kurrooa *Royle ex Benth. is an endangered plant species of medicinal importance. The medicinal property is attributed to monoterpenoids picroside I and II, which are modulated by temperature. The transcriptome information of this species is limited with the availability of few hundreds of expressed sequence tags (ESTs) in the public databases. In order to gain insight into temperature mediated molecular changes, high throughput *de novo *transcriptome sequencing and analyses were carried out at 15°C and 25°C, the temperatures known to modulate picrosides content.

**Results:**

Using paired-end (PE) Illumina sequencing technology, a total of 20,593,412 and 44,229,272 PE reads were obtained after quality filtering for 15°C and 25°C, respectively. Available (e.g., De-Bruijn/Eulerian graph) and in-house developed bioinformatics tools were used for assembly and annotation of transcriptome. A total of 74,336 assembled transcript sequences were obtained, with an average coverage of 76.6 and average length of 439.5. Guanine-cytosine (GC) content was observed to be 44.6%, while the transcriptome exhibited abundance of trinucleotide simple sequence repeat (SSR; 45.63%) markers.

Large scale expression profiling through "read per exon kilobase per million (RPKM)", showed changes in several biological processes and metabolic pathways including *cytochrome P450s *(*CYPs*), *UDP-glycosyltransferases *(*UGTs*) and those associated with picrosides biosynthesis. RPKM data were validated by reverse transcriptase-polymerase chain reaction using a set of 19 genes, wherein 11 genes behaved in accordance with the two expression methods.

**Conclusions:**

Study generated transcriptome of *P. kurrooa *at two different temperatures. Large scale expression profiling through RPKM showed major transcriptome changes in response to temperature reflecting alterations in major biological processes and metabolic pathways, and provided insight of GC content and SSR markers. Analysis also identified putative *CYPs *and *UGTs *that could help in discovering the hitherto unknown genes associated with picrosides biosynthesis.

## Background

*Picrorhiza kurrooa *Royle ex Benth. is a medicinally important endangered plant species of family Scrophulariaceae. The species is distributed between 3,000-5,000 m above mean sea level in the Himalayan region [1]. *P. kurrooa *is widely used in traditional as well as modern system of medicine for the treatment of liver disorders, fever, asthma and jaundice [2,3]. Indiscriminate and extensive harvesting and lack of organized cultivation has threatened the status of this plant in nature and is listed as "endangered species" by International Union for Conservation of Nature and Natural Resources [4]. Due to narrow distribution range, small population size and high use, the species appears among the 37 identified as top priority species for conservation and cultivation in western Himalaya.

The biological activity of *P. kurrooa *is attributed to the presence of iridoid glycosides mainly picroside I and picroside II. Studies have shown that temperature plays an important role in the biosynthesis and accumulation of picrosides [5]. A temperature of 15°C favored picrosides accumulation as compared to 25°C and this was in agreement with the expression of *1-deoxy-D-xylulose-5-phosphate synthase *(*DXS*) and *3-hydroxy-3-methylglutaryl-coenzyme A reductase *(*HMGR*), the genes associated with picrosides biosynthesis. However, the progress in unraveling the molecular response of *P. kurrooa *at these temperatures has been impeded by the dearth of transcriptomic resources. Transcriptome sequencing is an efficient way to understand global molecular response by the plant in response to a cue [6]. Expressed sequence tags (ESTs) played a significant role in accelerating gene discovery, expression analysis, improving genome annotation, identifying splice variants, and identification of molecular markers [7-9]. Transcriptome sequencing through next generation sequencing technology provides extensive data in much shorter time period with enormous depth and coverage to facilitate understanding of major change in the metabolic processes as well as contribute to comparative transcriptomics, evolutionary genomics and gene discovery [10-15]. Illumina genome analyzer based sequencing technology (Illumina, USA) yields huge amount of short reads with high coverage. Assembling such short reads is a challenging task, more so in the absence of reference sequences. A few bioinformatics tools have been developed for *de novo *assembly using short-read sequence data [16,17], which vary in their success and application, and depends upon data specific strategies.

The present study describes the first global analysis of *P. kurrooa *transcriptome under two temperature regimes, which would serve as a blueprint of gene expression profile. The work reports a strategy for *de novo *assembly of transcriptome using short-read sequence data generated by Illumina RNA-Seq method. Read per exon kilobase per million (RPKM) based comparative expression profiling study was done to systematically characterize the mRNAs at two temperatures and to identify the differentially regulated genes including those involved in picrosides biosynthesis. Data on *in silico *gene expression was validated by reverse transcriptase-polymerase chain reaction (RT-PCR) as well using a set of 19 genes.

## Results and Discussion

Transcriptome represents the expressed portion of the genome and offers an overall view of the transcribed genes. It is a powerful tool in gene discovery and in understanding the biochemical pathways involved in physiological responses. Various techniques such as microarray, serial analysis of gene expression and massively parallel signature sequencing emerged for high throughput gene expression profiling in the past and to allow for simultaneous interrogation of gene expression on a genome-wide scale [18]. However, these techniques are time consuming and become expensive, particularly for the analysis at global level. Also, biases are introduced by the inevitable cloning step [19].

With the advent of next generation sequencing technology, transcriptome analysis takes lesser time, cost and labour, and at the same time provides major sequence coverage and depth [8]. The technology has been used with success for the analysis of transcriptomes of several plant species including *Cajanus cajan *[20], *Arabidopsis thaliana *[21,22], *Medicago truncatula *[23], *Zea mays *[24], *Hordeum vulgare *[25], *Lycopersicum esculentum *[26], *Camellia sinenis *[27], and *Cicer arietinum *[28].

The present work was carried out on *de novo *transcriptome sequencing of *P. kurrooa*, *de novo *assembly of short reads, annotation of assembled sequences at 15°C and 25°C and validation of RPKM based expression analysis by RT-PCR using selected genes. *P. kurrooa *is a medicinally important and endangered plant species. Medicinal properties are attributed to the monoterpenoids, picroside I and picroside II, which are associated with hepatoprotective activity as one of the major activities [2].

### Reads generation and *de novo *sequence assembly

The *de novo *assembly of short reads without a reference genome still remains a challenge in spite of the development of many bioinformatics tools for data assembly and analysis [17,29]. Paired-end (PE) run of 36 cycles, for each of the leaf tissues collected from plants exposed to 15°C and 25°C, was performed on Illumina genome analyzer IIx platform (Illumina, USA). Bcl converter was used to produce the reads in qseq format of the PE run of genome analyzer. It contains reads, their coordinates, tile number and quality encoding. Since 3' ends of reads are prone to sequencing error, for every 36 bp read, only 33 bases (excluding the 3 bases at 3' end) were considered for further use. A total of 27,562,496 and 49,274,224 PE reads were generated at 15°C and 25°C, respectively. After performing quality filtering, a total of 20,593,412 and 44,229,272 PE reads were obtained for 15°C and 25°C, respectively. In total 64,822,684 PE reads were obtained.

In order to select the most appropriate k-mer size for considering *de novo *assembly, SOAPdenovo was run at different k-mer size ranging between19 to 29 mers, with read length of 33 bp [18]. The parameters recorded were the total transcripts obtained after assembling, average coverage, average transcript size, percentage of transcripts having length higher than 1,000 bp and highest transcript length. K-mer size of 23 mer emerged as the best choice for performing assembly, as it had a balance between over-represented and under-represented transcript numbers, coverage, maximum length obtained and average transcript length (Table 1). Total number of transcripts decreased linearly with the increment of k-mer size suggesting over-representation at lower k-mer and under-representation at higher k-mers. It was observed that the sequences assembled at higher k-mer were enriched for transcripts with higher coverage/higher expression. For assembly process, only those reads were considered that produced high frequency k-mer. PE module was used to perform more sensitive assembling, and utilized pair information and approximate distance between PE reads (200 bp). Gap filling was opted to produce longer scaffolds from the PE reads data, mapping into the contigs as well as gapped regions. For all the assembling steps, minimum length cut-off for assembled transcripts was set to 100 bp.

**Table 1:** Effect of k-mer size on assembling performance of transcriptome

k- mer	Average coverage	Average length (bp)	Maximum length (bp)	Total transcripts	Per cent transcripts above 1000 bp
19	76.6	421.23	7102	89,988	7.54
21	76.6	432.42	6873	83,774	8.94
23	76.6	439.55	5759	74,336	9.16
25	76.6	417.5	6350	62,526	8.24
27	77.8	379.85	5097	47,389	6.73
29	86.9	333.96	4441	26,733	4.59

The data from the two temperature conditions were assembled separately. For 15°C assembly, a total of 31,338 assembled transcripts with average length of 403.87 bp and average coverage of 64.68 times was obtained (Table 2). Total 2,029 assembled transcripts (6.48%) had sequence length longer than 1,000 bp with the longest assembled transcript of 5,326 bp. Similarly for 25°C, 63,718 assembled transcript sequences were obtained with 434.39 bp average length and average coverage of 71.26. Total 4,988 sequences (7.82%) were longer than 1,000 bp with maximum sequence length of 5,210 bp. The difference in the number of assembled transcripts generated for 15°C and 25°C could be the result of the stress response of the plants at high temperature [30]. It is also likely that such differences in the number of assembled transcripts could be due to the technological noise that might have crept in at some stage, as reported by previous work [31]. Considering the quality of assemblies produced at two different temperatures, it appeared that increased read data could enhance the total coverage, average contig length and percentage of transcripts longer than 1 kb. Therefore, PE reads of the two lanes (15°C and 25°C) were combined, retaining the PE information. Combined *de novo *assembly was carried out with 64,822,684 filtered reads in PE form (Table 2). With this approach, a total of 74,336 transcripts were obtained, of which 9.16% were above 1,000 bp, yielding higher average coverage of 76.6, average length of 439.5 bp and maximum length of 5,759 bp. Therefore, total 74,336 transcripts, generated from pooled reads, made the final representatives for assembled sequences in this study. The related read and sequence (contigs) data have been deposited at National Centre for Biotechnology Information (NCBI) in the Short Read Archive (SRA) database and Transcriptome Shotgun Assembly (TSA) database under the accession numbers SRA048843.1 and JR808536-JR842818, respectively. Those sequences, which were either less than 200 bp or had a stretch of "N" > 14 nucleotides were not accepted by TSA and hence were presented in Additional file 1 and Additional file 2 which contains complete collection of assembled sequences.

**Table 2:** Summary of transcriptome data generated on Illumina genome analyzer IIx for leaf tissue of *P. kurrooa*.

	15°C	25°C	Total/Pooled
Total number of paired-end reads	27,562,496	49,274,224	76,836,720
Number of reads obtained after quality filtering	20,593,412	44,229,272	64,822,684
Number of assembled transcripts	31,338	63,718	74,336
Average length of transcripts (in bp)	403.87	434.39	439.5
Average coverage (%)	64.68	71.26	76.6

The table shows assembly quality when assembled for reads generated at 15°C and 25°C, separately and by pooling the reads obtained at the two temperatures together. The best contigs were found for the pooled data having higher coverage and higher average transcript length

### Homology search and sequence clustering

Set of assembled sequences contained several sequences sharing similarity, causing over-representation of data for total transcript sequence measurement. Such redundancy and over-representation can be reduced by finding similar sequences either through merging them or by using a single representative sequence instead. This was done by applying sequence similarity based clustering. After performing hierarchical clustering with TIGR Gene Indices clustering tools (TGICL) using Contig Assembly Program (CAP3) and Cluster database at high identity with tolerance (CD-HIT), with minimum 90% similarity cut off, the number of unique assembled transcripts reduced from 74,336 to 72,220.

Analysis of sequences, obtained after clustering, was done for homology search against protein sequences at non-redundant (NR) databases at NCBI by BLASTX with cut off E-value of 10^-5^. For pooled read assembled transcript sequences, significant BLAST hits were found for a total of 42,598 sequences while no hit was found for 29,622 sequences.

Another clustering step was carried out for sequences which returned significant BLAST hits. Sequences with no apparent significant identity among themselves might belong to the different parts of the same gene or may represent the isoforms. Counting them as separate transcripts would only inflate the number of unique genes. Therefore, all those transcripts were searched that exhibited significant hits and shared the best hits to the same reference sequence. A set of local scripts was written to scan for all those assembled transcript sequences that returned a common best hit and common reference gene but differed in their location. All such transcripts were clustered together and assumed as the members of associated reference sequence/gene represented. This step reduced the total number of transcripts with significant BLAST hits, from 42,598 to 28,403. Therefore, the number of actual unique genes is expected to be much lower than the total transcript sequences coming out of any *de novo *assembling tool. Such clustering approach was applied in order to get the correct number of unique genes represented by the assembled transcripts sequences, which could be over-represented otherwise. A complete detail of grouped sequences has been made available in Additional file 3. Related materials and associated annotations for sequences representing the group sequences as the best one is available at the URL: http://scbb.ihbt.res.in/Picro_information. Remaining 29,622 transcript sequences, with no significant homologous reference sequence, too could display the above mentioned clustering property and their total number might go lower than the observed. However, in this part of the study, ORFs were derived in six frames of these unknown sequences and looked for possible functional domains across the conserved domain databases. Interestingly, for 1,225 sequences, a few significant conserved domains were identified. Some of the highly represented domains were Fibronectin attachment protein (FAP), large tegument protein, extensin-like, formin homology region 1, TT-viral orf1, cysteine-rich transmembrane module stress tolerance and fibrillarin (Additional file 4). These hitherto unknown sequences might render the function characteristic to the domain.

### Validation of assembled sequences against the ESTs of *P. kurrooa*

The assembled sequences obtained from pooled reads were validated by sequence based alignments against ESTs of *P. kurrooa *submitted at NCBI dbEST by our group. For 500 submitted ESTs (Additional file 5; this has accession numbers of the submitted ESTs), BLASTN analysis against the assembled transcripts was performed with an E-value threshold of 10^-05^. The sequences were analyzed for mis-assemblies and manually checked for all alignment conditions. Besides this, manual assessment of alignment for all transcripts with their BLASTX hits against protein NR databases at NCBI was also performed. Significant hits were observed for 417 sequences (83.4%), while no hit could be obtained for 83 ESTs from the assembled transcript set. Also, most of the assembled transcript sequences were found to be aligned correctly and in continuous form, with average identity of 96.35%, suggesting good assembly quality. The unmappable unigenes in *P. kurrooa *might include fusion transcripts, relatively short and low quality singletons, UTR sequences far from the translation start or stop sites (> 1,000 bp), and those having incomplete coverage by the genome. It has been reported that even in *A. thaliana *around 13% of the ESTs could not be aligned to the predicted genes [32] and in human only 64% of the reads could be mapped to the RefSeq database of well annotated human genes [33]. Detailed information regarding this part of analysis is mentioned at URL http://scbb.ihbt.res.in/Picro_information.

### Utilization of transcriptome data for analysis of guanine-cytosine (GC) content and identification of simple sequence repeats (SSRs) markers

Next generation sequencing offered an opportunity for the analysis of GC content among unigenes and expanded the scope for molecular markers such as SSRs. GC content gives important indication about the genes and genomic composition including evolution, gene structure (intron size and number), gene regulation and is an indicator of stability of DNA [34]. Average GC content of *P. kurrooa *transcripts was 44.6% (Additional file 6), which is in range of GC levels of coding sequences in dicots (44-47%) [35].

SSRs or microsatellites markers have diagnostic and functional significance, and have been usually associated with functional and phenotypic variations. SSRs are multi-allelic in nature, reproducible, highly abundant, cover the genome extensively and exhibit co-dominant inheritance. Transcriptome SSR markers exhibit high interspecific transferability [36]. Due to the limitation of genomic data available, EST databases have been increasingly screened for development of genic SSRs [37-41]. *P. kurrooa *is a cross pollinated species and hence the seed raised population will have variability. Variability in vegetative growth as well as for picrosides content of wild populations of *P. kurrooa *has also been reported [42]. Also, considerable variation exists in picrosides content for plants collected from different locations [43]. The identification of SSRs in *P. kurrooa *will help in distinguishing closely related individuals and will also provide useful criteria for enriching and analyzing variation in the gene pool of the plant.

Assembled transcript sequences of *P. kurrooa *were analyzed for these SSRs (Additional file 7). A total of 1,562 SSRs were identified in the assembled transcripts. The trinucleotide SSRs have been observed to be the most prevalent group of markers (45.63%) with highest occurrence of GAA, TGG, CCA, AGA, and TCA repeats followed by mononucleotide (35.25%) and dinucleotide (21.29%) SSRs. Most prevalent mononucleotide was poly-A while highest prevalent dinucleotide SSRs were poly-AG and poly-TC. The observed prevalence of poly-A could be due to the presence of poly-A tails of RNA sequences. Only a small fraction of tetra and penta SSRs were identified (Additional file 7). In general, trinucleotide SSRs are the most common ones as compared to dinucleotides or tetranucleotides [40,41].

### Functional annotation and classification of *P. kurrooa *transcriptome

**Figure 1:**
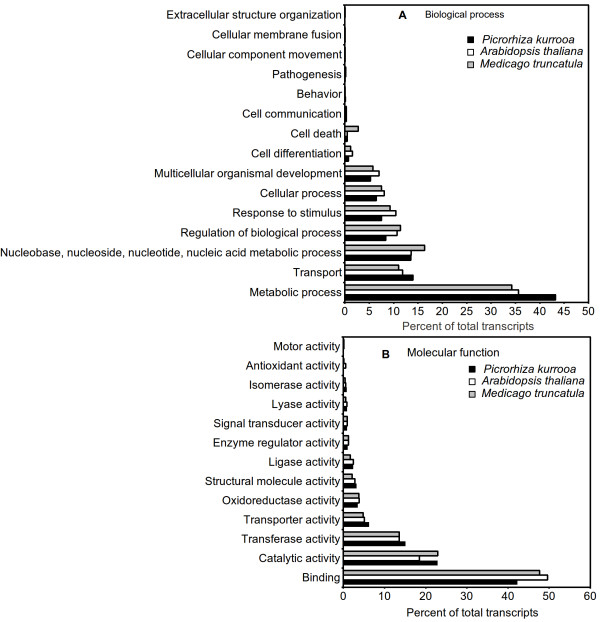
**Gene Ontology classification of the *P. kurrooa *transcriptome and its comparison with *A. thaliana *and *M. truncatula***. Gene Ontology (GO) term assignments to *P. kurrooa *unigenes, based on significant plant specific GO slims, were summarized into two main GO categories: biological process (**A**), and molecular function (**B**).

For functional annotation of *P. kurrooa *transcriptome, transcripts were compared against the NR protein sequences available at UniProt database using BLASTX algorithm with E-value threshold of 10^-01^. The associated hits were searched for their respective Gene Ontology (GO), Kyoto Encyclopedia of Genes and Genomes (KEGG) and Enzyme Commission Codes (EC) for each query sequence and the highest bit score hit was selected. GO terms are derived from dynamic controlled vocabularies or ontologies that can be used to describe the function of genes and gene products. Annotation against GO database yielded significant annotation for 31,959 out of 72,220 assembled sequences, representing the best possible hits. These genes were further classified into two major categories namely, biological process and molecular function using plant specific GO slims that provide a broad overview of the ontology content. Functional classification of *P. kurrooa *transcripts in biological process category (Figure 1A) showed that metabolic process, transport, regulation of biological processes, response to stimulus and cellular process were among the highly represented groups indicating that the plant is undergoing rapid growth and extensive metabolic activity. Genes involved in DNA binding, catalytic and transferase activity were highly represented in molecular function category (Figure 1B) indicating dominance of gene regulation, signal transduction and enzymatically active processes. Genes involved in other important biological processes such as cell differentiation, communication, transport were also identified through GO annotations. A comparison for distribution of transcripts across various GO slim categories, between *A. thaliana*, *P. kurrooa *and *M. truncatula *showed no major differences between the ranks of GO slim categories (Figure 1A, B). However, comparison for distribution of top occurring GO terms instead of GO slim categories between *P. kurrooa *and *A. thaliana *suggested differential distribution of transcripts between various GO categories (Additional file 8).

**Figure 2:**
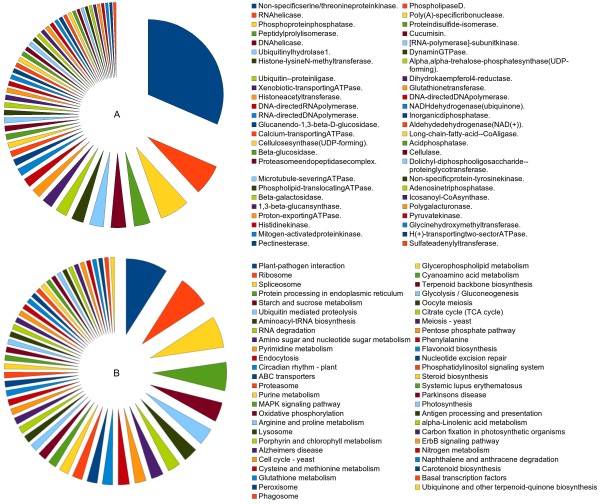
**Functional characterization and abundance of *P. kurrooa *transcriptome for enzyme classes (A), and KEGG pathways (B)**. *P. kurrooa *transcripts were classified in top 50 abundant enzyme classes and KEGG pathways; area under each pie represents the value in per cent.

Best EC classification was obtained for 14,630 assembled sequences, whereas associated KEGG classification was obtained for 15,560 assembled sequences. Figure 2A lists the top 50 abundant enzyme classes observed for *P. kurrooa *transcriptome. Interestingly, a large amount of assembled transcripts belonged to serine/threonine protein kinase enzyme class alone (14.6%). Besides this, Figure 2B displays top 50 KEGG pathways represented by the assembled transcriptome sequences. Highest number of sequences belonged to plant-pathogen interaction pathways (6.13%) followed by ribosome, spliceosome, protein processing and endoplasmic reticulum, starch and sucrose metabolism, ubiquitin mediated proteolysis, aminoacyl-tRNA biosynthesis, RNA degradation and so on. Highest represented groups included many pathways associated with housekeeping processes as well as plant development and secondary metabolism.

### Transcriptome analysis suggests modulation of major plant processes at two temperatures

**Figure 3:**
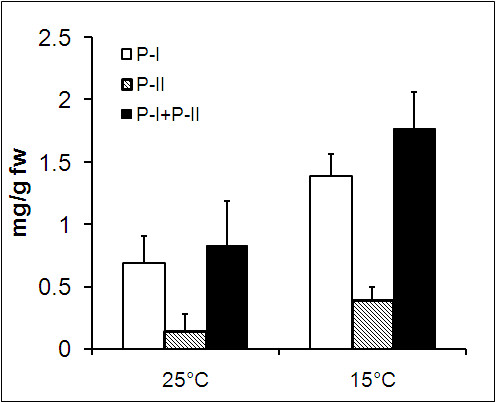
**Effect of temperature on picrosides content**. Leaf tissue collected from *P. kurrooa *plants kept at 15°C and 25°C were ground in liquid nitrogen and the picrosides were extracted for analysis on Ultra Performance Liquid Chromatography system using BEH workflow shield C18 (1.7 μm particles, 2.1 × 100 mm) analytical column. Data are average of four separate biological replicates with error bars as standard deviation.

**Figure 4:**
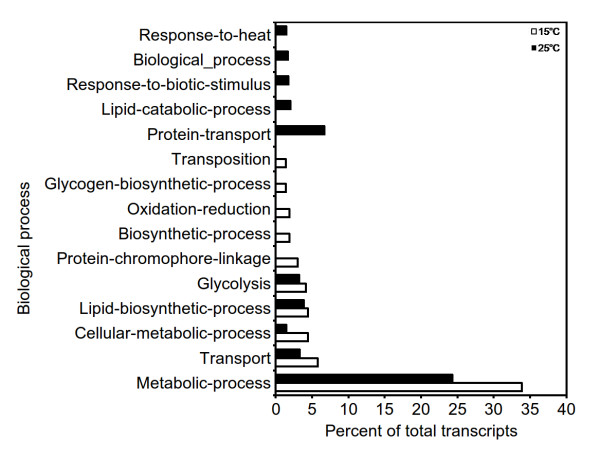
**Distribution of *P. kurrooa *unigenes across various biological process categories exhibiting 2 fold or higher expression at 15°C and 25°C [based on a set of plant specific Gene Ontology (GO) terms]**. For some transcripts, the daughter GO terms could not be assigned, making them to retain higher hierarchy GO terms or GO slim terms.

**Figure 5:**
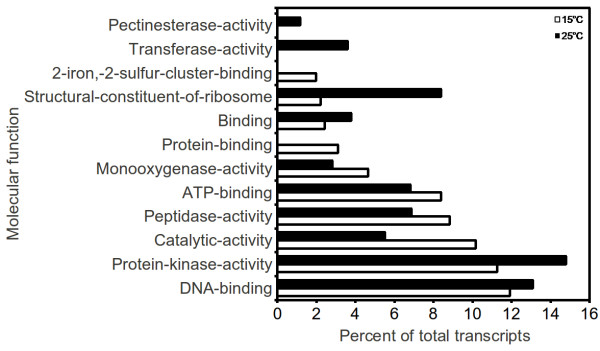
**Distribution of *P. kurrooa *unigenes across various molecular function categories exhibiting 2 fold or higher expression at 15°C and 25°C [based on a set of plant specific Gene Ontology (GO) terms]**. For some transcripts, the daughter GO terms could not be assigned, making them to retain higher hierarchy GO terms or GO slim terms.

**Figure 6:**
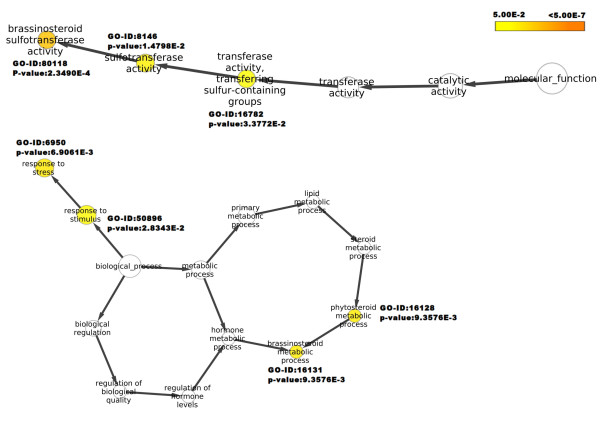
**Significantly enriched functional categories observed for the genes over-expressed at 25°C as compared to those at 15°C in *P. kurrooa***. The *P. kurrooa *transcripts were analyzed using BiNGO, where colored nodes represent the significantly enriched GO terms with their statistical significance. Node size is proportional to the number of transcripts in each category. Different color shade represents different significance level (white - no significant difference; color scale, yellow, p-value = 0.05; orange, p-value < 0.0000005).

One of the goals of transcriptome sequencing was to compare transcripts at 15°C and 25°C, the temperatures which modulate picrosides content [5]. Our data was in line with the previous work that showed higher picrosides content at 15°C as compared to those at 25°C (Figure 3). Genome wide analysis of gene expression at two temperatures was assessed using RPKM, where read-counts of a particular transcript represent expression level [44]. This approach was effective in detecting even scarcely expressed transcripts and was reported to be independent of prior knowledge of gene model, making it a natural choice to measure expression in the absence of known gene models and microarray chips. Considering dissimilar sequence clustering to contain over-representation, GO annotation for 19,769 unique genes was obtained along with their RPKM values at the two temperatures. Based on fold increment at 15°C, these genes were grouped into six different expression classes ranging from > 10 fold increment to 0.5 fold or lower. Highest number of genes fell into the group with minimum change in expression. A detailed breakup for each of such categories along with top 20 GO categories for molecular function as well as biological process is listed in Additional file 9. Molecular function groups representing monooxygenase activity, 2-iron, 2-sulfur cluster binding, cobalamin binding, beta lactamase activity, aminobutyraldehyde dehydrogenase activity, purine transmembrane transporter activity and metal ions like iron and copper binding activities were found to be over-expressed at 15°C. Under the biological process groups, those associated with various biosynthesis and transport processes such as zinc and ammonium transport and protein chromophore linkage were prominent at 15°C. Figure 4 and 5 represent the top 10 biological process and molecular function categories, respectively, present at two fold or higher expression groups at 15°C and 25°C. As can be seen from these diagrams, response to heat, response to biotic stimulus and lipid catabolic process were exclusively represented at 25°C. While at 15°C, categories for redox, glycogen biosynthetic process, biosynthetic processes and protein-chromophore linkage were exclusively represented. Under the molecular function categories, monooxygenase activities, peptidase activities, catalytic activities, 2-sulfur, 2-iron cluster binding and protein binding activities were found to be more pronounced at 15°C, while pectinesterase and protein kinase activities were predominant at 25°C. A further enrichment analysis for functional categories pointed out that transcripts associated with response to stress, response to stimulus, phytosteroid metabolic process and brassinosteroid (BR) metabolic process were significantly enriched in the group having two fold or higher expression at 25°C (Figure 6). BRs are a group of plant steroidal hormones that regulate various aspects of plant growth and development, including cell elongation, photomorphogenesis, xylem differentiation, seed germination [45], and adaptation to abiotic and biotic environmental stresses [46]. BRs promote tolerance in plants against a wide range of stresses, including heat, cold, drought and salinity, possibly through up-regulating the expression of stress related genes [47]. Compared to the expression at 15°C, up-regulation of stress responsive transcripts is suggestive of *P. kurrooa *to be under stress at 25°C. The above described global analysis of gene expression provided comprehensive dataset with each gene represented by its absolute expression level at the two temperatures. Several processes such as metabolic processes, cellular processes, transport processes (Figure 4), and catalytic processes (Figure 5) were equally represented at both the temperatures indicating the importance of these processes in plant growth and survival; although different set of genes in these processes might determine the response of plant to temperature change. Transcripts associated with the processes involving lipid metabolism were highly enriched at 25°C (Figure 4), suggesting a change in lipid profile. Indeed, temperature has been shown to modulate lipid profile in plant [48].

Concomitant modulation of several plant processes suggests involvement of transcription factors (TFs) for coordinated regulation of gene expression. TFs are sequence specific DNA-binding proteins that interact with the promoter regions of target genes and modulate gene expression. These proteins regulate gene transcription depending upon tissue type and in response to internal signals, for example plant hormones, and to external signals such as temperature, UV light, pathogen attack, and drought. In *P. kurrooa*, 6,305 transcript sequences exhibited homology with TF families, which were reduced to 2,500 after dissimilar sequence clustering (Additional file 10). The most abundant TF families observed were for *C3H*, *PHD*, *MADS*, *bHLH *and *MYB*-related family (Additional file 11). Of the 2,500 transcription factors, 5.36% exhibited 2-fold or higher abundance enrichment at 15°C (Additional file 12). The most abundant TFs in this group were *MYB*-related, *FAR1*, *FHA*, *HB*, *bHLH*, *Orphans*, *C3H*, *C2H2*, *MADS*, *G2-like*, *NAC*, *SNF2 *and *WRKY*. Of the total TFs, 16.9% exhibited abundance increment two fold or above at 25°C and belonged to *C3H*, *MADS*, *bHLH*, *PHD *and *FAR1 *family.

The above mentioned TFs have been associated with varied processes. For example, members of C3H family are involved in embryogenesis [49], whereas PHD proteins are found in nucleus and regulate chromatin-mediated transcription [50]. Most members of MADS family TFs are involved in the regulation of flower-related physiological and developmental processes [51], whereas members of bHLH are involved in controlling cell proliferation and in the development of specific cell lineages [52]. FAR1, yet another family of TFs, is involved in phytochrome signaling [53].

It was interesting to note that global gene expression analysis exhibited modulation of processes which were responsive to heat, responsive to biotic stimulus, lipid catabolic process and glycogen biosynthetic process (Figure 4) at 25°C as compared to 15°C. Members of TFs families such as bHLH, WRKY, MYB, AP2/EREBP [54-57] that are known to regulate the above processes, also exhibited modulation (Additional file 12) in accordance with the said transcripts, suggesting their role in regulating the mentioned processes. *P. kurrooa *is a plant of high altitude temperate region which does not tolerate a continuous temperature greater than 20°C for a longer period of time [58]. A systematic analysis of these transcription factors would open door for imparting tolerance to *P. kurrooa *at high temperature (25°C). It would also be worthwhile to study, how such a temperature mediated transcriptomic adjustment affects picrosides biosynthesis.

### Pathways associated with picrosides biosynthesis exhibit temperature dependent modulation

**Figure 7:**
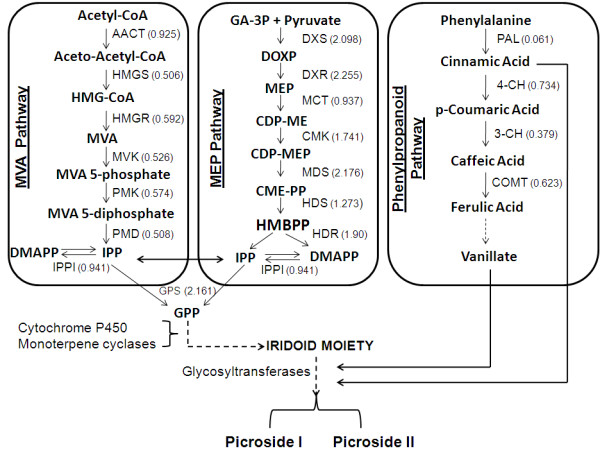
**Picrosides biosynthetic pathway in *P. kurrooa *(adapted from Kawoosa et al. and Dixon and Paiva **[5,59]). Picrosides are iridoid glycosides derived from cyclization of geranyl pyrophosphate (GPP) to iridoid moiety. Glucose and cinnamate/vanillate convert iridoid into picroside I and picroside II. These steps involve series of hydroxylation and glycosylation reactions catalyzed by cytochrome P450 and glycosyltransferases. GPP can be derived from mevalonate (MVA) or 2-C-methyl-D-erythritol 4-phosphate (MEP) pathway. Enzymes of MVA pathway are as follows: AACT, acetyl-CoA acetyltransferase; HMGS, 3-hydroxy-3-methylglutaryl-CoA synthase; HMGR, 3-hydroxy-3-methylglutaryl-coenzyme A reductase; MVK, mevalonate kinase; PMK, phosphomevalonate kinase; PMD, diphosphomevalonate decarboxylase. Enzymes of MEP pathway are DXS, 1-deoxy-D-xylulose-5-phosphate synthase; DXR, 1-deoxy-D-xylulose-5-phosphate reductoisomerase; MCT, 2-C-methyl-D-erythritol 4-phosphate cytidylyltransferase; CMK, 4-diphosphocytidyl-2-C-methyl-D-erythritol kinase; MDS, 2-C-methyl-D-erythritol 2,4-cyclodiphosphate synthase; HDS, 4-hydroxy-3-methylbut-2-enyl diphosphate synthase; HDR, 4-hydroxy-3-methylbut-2-enyl diphosphate reductase. Isopentenyl pyrophosphate isomerase (IPPI) catalyzes the isomerisation of dimethylallyl pyrophosphate (DMAPP) to IPP whereas conversion of IPP to geranyl pyrophosphate (GPP) is catalyzed by geranyl pyrophosphate synthase (GPS). Enzyme of phenylpropanoid pathway involved in biosynthesis of cinnamate are PAL, phenylalanine ammonia-lyase; 4-CH, cinnamic acid 4-hydroxylase; 3-CH, p-coumarate 3-hydroxylase; COMT, caffeoyl-CoA 3-O methyltransferase. Solid arrows indicate known steps, whereas broken arrows represent unknown intermediates and enzymes. Numerals in parenthesis indicate fold change in gene expression at 15°C as compared to 25°C based on reads per exon kilobase per million (RPKM) values (detailed RPKM values are shown in Figure 8 and Additional file 13).

Since the two temperatures targeted in the present work modulated picrosides content, it was of interest to analyze various genes associated with picrosides biosynthesis. Picrosides are terpenoids with an iridoid skeleton of monoterpene origin. Depending upon the functional-group moieties, picrosides are classified as picroside I and picroside II. Picroside I has cinnamate moiety, whereas picroside II has vanillate moiety. The cinnamate and vanillate moieties are derived from PP pathway (Figure 7). Synthesis of cinnamate requires the action of phenylalanine ammonia-lyase (PAL) on phenylalanine whereas caffeoyl-CoA 3-O methyltransferase (COMT) is the key enzyme for vanillate biosynthesis [59].

Iridoid moiety is derived from geranyl pyrophosphate (GPP) (Figure 7). GPP is synthesized by sequential head to tail addition of isopentenyl pyrophosphate (IPP) and its allelic isomer dimethylallyl pyrophosphate (DMAPP) [60]. Cytosolic MVA pathway and the plastid localized MEP pathway synthesize IPP and DMAPP [60-64], with cross talks between these two pathways [65,66]. Thus MVA, MEP and PP are regarded as central pathways for the synthesis of picrosides. MVA pathway starts from the condensation of acetyl-CoA [67,68], whereas MEP pathway needs pyruvate and glyceraldehyde 3-phosphate [69,70]. Biosynthesis of picrosides involves synthesis of iridoid moiety from GPP through series of oxidation and cyclization steps followed by the condensation of glucose moiety and cinnamate/vanillate with iridoid unit (Figure 7).

Analysis of MVA, MEP and PP pathways have general implications as well. These are involved in the biosynthesis of large number of secondary metabolites including those having commercial implications. These compounds include taxol [71,72], artemisinin [73], β-carotene [74], α-tocopherol [75], vincristine, vinblastine and coumarins [76]. These pathways play important roles in growth and development including secondary metabolism, and hence identification of major regulatory steps would be key to modulate plant performance and secondary metabolism, if need be.

**Figure 8:**
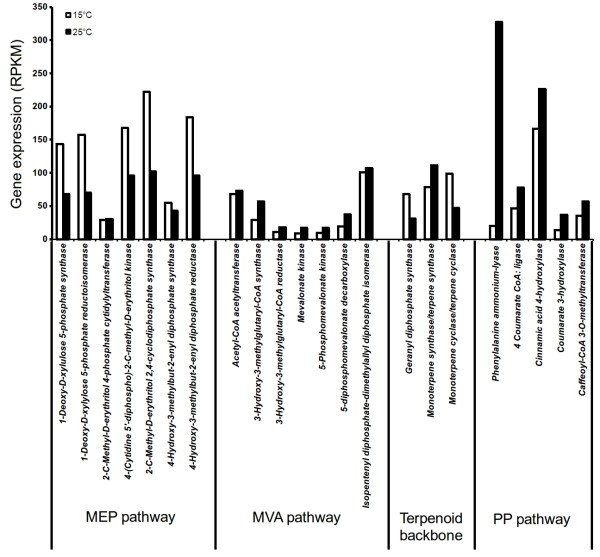
**Histogram showing transcript abundance of various pathways (Figure 7 details the pathway) associated with picrosides biosynthesis at 15°C and 25°C based on reads per exon kilobase per million (RPKM) values**. Mevalonate (MVA), 2-C-methyl-D-erythritol 4-phosphate (MEP) and phenylpropanoid (PP) are central pathways for picrosides biosynthesis.

Using BLAST analysis against the UniProt and KEGG databases, various genes associated with MVA, MEP and PP pathways were identified. RPKM-based expression showed 2 fold increase for several genes of MEP pathways at 15°C as compared to those at 25°C (Figure 8). Data was in agreement with the data on picrosides content that showed its increased accumulation at 15°C (Figure 3). While a previous work on *P. kurrooa *also showed a positive correlation between *Pkdxs *(a gene of MEP pathway) and picrosides accumulation [5], the present work detailed on all the genes of MEP pathway highlighting their importance in picrosides accumulation (Figure 8).

RPKM data showed up-regulation of various genes of MVA and PP pathway at 25°C as compared to those at 15°C (Figure 8, Additional file 13). The accumulation of picrosides decreased at 25°C as compared to 15°C, whereas various genes of PP pathway exhibited up-regulation at 25°C. Since PP pathway is important in supplying cinnamate and vallinate for picrosides biosynthesis, an up-regulation of various genes of the pathways were envisioned at 15°C as compared to 25°C. However the results were opposite, suggesting rerouting of the metabolites towards the synthesis of other metabolites at 25°C. And at 15°C, the observed expression of genes of the MVA and PP pathway might be sufficient enough to meet the requirement of cinnamate and vanillate. In fact, increased activity of *PAL *(a gene of PP pathway) in response to thermal stress was considered as an acclamatory response of cells to heat stress in *Citrulus vulgaris *[77]. Expression of various genes of PP pathway including *PAL *is regulated by TF family LIM. LIM proteins have conserved cysteine-histidine rich, zinc-coordinating domain consisting of two zinc fingers repeated in tandem. In transgenic tobacco, down-regulating the expression of LIM proteins through antisense approach lowered the expression of various genes of PP pathway [78]. In *P. kurrooa*, RPKM based expression analysis showed that the expression of *LIM *was up-regulated at 25°C (Additional file 12) suggesting its role in regulating PP pathway. Thus at higher temperature, up-regulation of MVA and PP pathways could have a role in temperature stress acclimation also.

### Transcriptome data identifies *cytochrome P450 *(*CYPs*) and *glycosyltransferases *(*GTs*) as a source of hitherto unknown genes involved in the biosynthesis of picrosides

In *P. kurrooa*, the intermediates and enzymes involved in cyclization of GPP for the synthesis of iridoid moiety and later its condensation with glucose and cinnamate/vanillate moieties are yet to be deciphered. *In vivo* tracer studies in *Catharanthus roseus *and *Lonicera morrowii *showed that iridoid is synthesized by cyclization of 10-oxogeranial to yield iridoial [79]. This is subsequently converted into iridoid compounds via iridotrial intermediate, and involves multiple oxidation/hydroxylation and glycosylation reactions. Most of the oxidative reactions, including hydroxylations, epoxidation, dealkylation, dehydration and carbon-carbon bond cleavage are catalyzed by CYP group of enzymes [80], whereas glycosylation reactions are catalyzed by GTs. Therefore, it would be relevant to discuss CYPs and GTs in the transcriptome of *P. kurrooa*.

CYPs are membrane bound hemoproteins involved in array of pathways in primary and secondary metabolism. Some of the example of CYPs include lauric acid hydroxylase, limonene-3-hydroxylases (CYP71D13 and CYP71D15), (+)-menthofuran synthase, geraniol hydroxylase, camphor-6-exo-hydroxylase, cinnamate 4-hydroxylase (4-CH), flavonoid 3'-hydroxylase, flavones synthase 2- berbamunine synthase, tyrosine N-hydroxylase. Based on phylogenetic studies, plant CYPs can be divided into 10 separate clans that cover the current 61 families [81].

**Figure 9:**
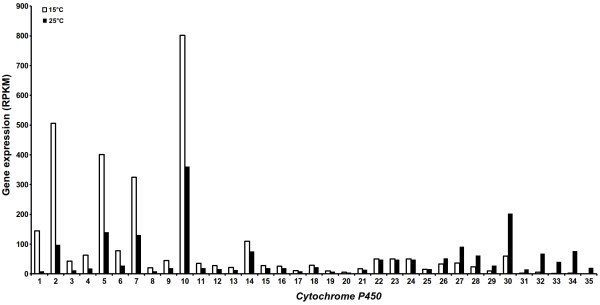
**Reads per exon kilobase per million (RPKM) values based gene expression of annotated *cytochrome P450s *(*CYPs*) in *P. kurrooa *transcriptome**. A total of 33 unigenes were annotated as putative *CYPs *in *P. kurrooa *transcriptome. Their expression was studied at 15°C and 25°C based on RPKM values. Numerals 1-33 represent 33 *CYPs *in the dataset, where 4, 6, 8, 9, 12-16, 18, 21, 23-24, 27-28, 30 were classified as putative *CYPs*. Details of other numerals are as follows 1, *flavonoid 3'-hydroxylase*; 2, *CYP92A46*; 3, *flavonoid 3'-hydroxylase*; 5, *cytochrome P450 NADPH-reductase*; 7, *CYP72A57*; 10, *CYP72A11*; 11, *CYP721A1*; 17, *coniferaldehyde 5-hydroxylase*; 19, *ABA 8'-hydroxylase CYP707A1*; 20, *CYP707A1*; 22, *cytochrome P450 monooxygenase CYP83G2*; 25, *cytochrome P450 monooxygenase CYP72A59*; 26, *cytochrome P450 monooxygenase CYP83E8*; 29, *cytochrome P450 CYP85A1*; 31, *CYP83B1*; 32, *CYP71AT2v2*; and 33, *cytochrome P450 fatty acid omega-hydroxylase*. Details on accession number and BLAST are mentioned in Additional file 14.

Monoterpenes, sesquiterpenes and diterpenes are intimately associated with CYPs. For example, l0-hydroxylation of geraniol and nerol has long been known to be a CYP function [82]. Allylic hydroxylation of cyclic monoterpenes by CYPs is well documented. Such reactions are known to occur in the hydroxylation of limonene [83], pinene [84], sabinene [85], camphor [86], abietin [87] and terpeniol [88]. All the CYPs associated with monoterpenes are divided into CYP71 clan. In the present study, 33 unigenes annotated as putative *CYPs *were identified based on highest bitscore and E-value (Figure 9 and Additional file 14). Sixteen of these *CYPs *were identified as putative *CYPs*. Seventeen *CYPs *exhibited homology with *flavonoid 3'-hydroxylase*, *CYP92A46*, *cytochrome P450 NADPH-reductase*, *CYP72A57*, *CYP72A11*, *CYP721A1*, *coniferaldehyde 5-hydroxylase*, *ABA 8'-hydroxylase CYP707A1*, *CYP707A1*, *cytochrome P450 monooxygenase CYP83G2*, *cytochrome P450 monooxygenase CYP72A59*, *cytochrome P450 monooxygenase CYP83E8*, *cytochrome P450 CYP85A1*, *CYP83B1*, *CYP71AT2v2*, and *cytochrome P450 fatty acid omega-hydroxylase*. Ten *CYPs *showed more than two fold increase in the expression and 8 *CYPs *exhibited down-regulation, respectively at 15°C as compared to at 25°C. Increased picrosides content (Figure 3) and up-regulation of *CYPs *at 15°C suggested these to be the possible candidates associated with picrosides biosynthesis through their possible role in cyclization of GPP and iridoid moiety as indicated in Figure 7.

GTs constitute a large family of enzymes that catalyze transfer of glycosyl group from activated sugars [activation is achieved after addition of nucleoside diphosphate e.g., uridine diphosphate (UDP) sugars] to aglycone acceptor molecules, and regulate their bioactivity, solubility and transport [89]. GTs are grouped into 69 families based on the substrate recognition and sequence relatedness, of which family 1 is the largest and is over-represented by uridine diphosphate glycosyltransferases (UGTs). UGTs use UDP-glucose as the donor in the GT catalyzed reactions [90]. Therefore, the reactions involving transfer of glucose utilize UGTs. UGTs have 44-amino acid C-terminal signature motif designated as PSPG box and are encoded by large multigene families, sometime comprising several hundred of genes. For example, family 1 UGTs are encoded by 120 *UGT *genes in *A. thaliana *and by 165 *UGTs *in *M. truncatula *[89]. The *UGT *superfamily in higher plants is thought to encode enzymes that glycosylate a broad array of aglycones, including plant hormones, all major classes of plant secondary metabolites, and xenobiotics such as herbicides [91]. Picrosides are present as 1-*O*-glucosides and hence an analysis of *UGTs *would be central to identify the gene associated with the glycosylation of iridoid moiety.

**Figure 10:**
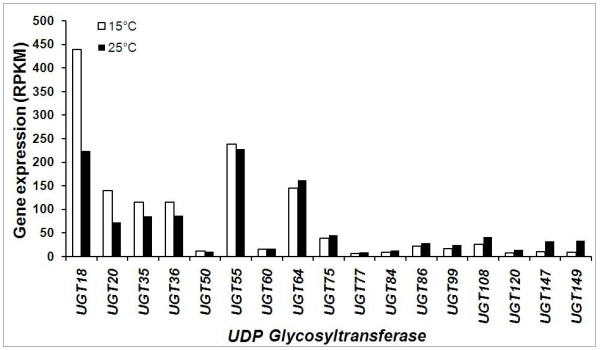
**Reads per exon kilobase per million (RPKM) values-based expression analysis of *uridine diphosphate glycosyltransferases *(*UGTs*) in *P. kurrooa *transcriptome**. Expression of 17 *UGTs *was studied at 15°C and 25°C. Details of the corresponding contigs, accession number and BLAST are listed in Additional file 15.

BLAST search identified 154 unigenes encoding GTs, out which 17 encoded for UGTs in *P. kurrooa *(Figure 10, Additional file 15). Expression of these 17 *UGTs *through RPKM analysis showed that 2 *UGTs *are up-regulated and 4 were down-regulated at 15°C as compared to those at 25°C. Eleven UGTs showed no significant change in expression at the two temperatures. Up-regulation of picrosides at 15°C vis-à-vis up-regulation of 2 *UGTs *suggests these to be the possible candidates associated with picrosides biosynthesis.

### Experimental validation of RPKM data by reverse transcriptase-polymerase chain reaction (RT-PCR)

**Figure 11:**
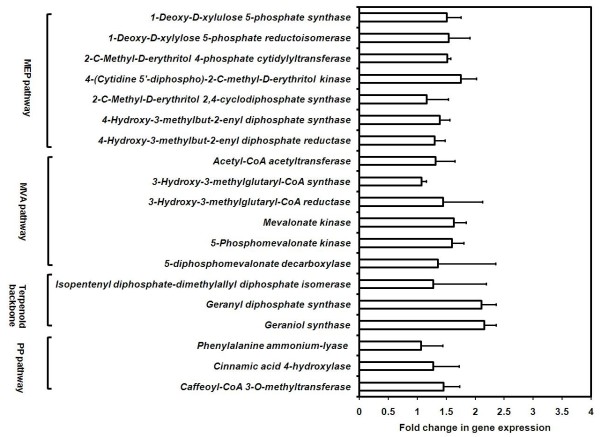
**Effect of temperature on expression of 19 genes of mevalonate (MVA), 2-C-methyl-D-erythritol 4-phosphate (MEP) and phenylpropanoid (PP) pathways (pathways associated with picrosides biosynthesis) as analysed by reverse transcriptase-polymerase chain reaction (RT-PCR)**. Total RNAs were extracted from the leaf tissue of plants exposed to 15°C and 25°C and changes in the abundance of transcripts were analyzed by RT-PCR using gene specific primers. Data shows fold increase in the transcript levels of putative genes at 15°C as quantified by measuring integrated density value (IDV) of amplicon. Data represents average of three separate biological replicates with error bars as standard deviation. Expression of *26 s rRNA *was used as control [92]. Additional file 16 details on primers and the PCR conditions used in RT-PCR.

The expression profiles obtained through RPKM values were experimentally validated through RT-PCR using 19 genes belonging to MEP, MVA and PP pathways. RT-PCR data showed that all the 7 genes of MEP pathway showed up-regulation at 15°C and the similar trend was obtained by RPKM data as well (Figure 8, Figure 11).

RT-PCR showed up-regulation of *HMGR*, *mevalonate kinase *(*MVK*) and *phosphomevalonate kinase *(*PMK*) of MVA pathway and *COMT *of PP pathway at 15°C. However, RPKM data exhibited up-regulation of these genes at 25°C. Expression of *acetyl-CoA acetyltransferase *(of MVA pathway) did not exhibit any significant variation at the two temperatures as evidenced by RT-PCR and RPKM data. Expression of *GPS *(Figure 8) was prominent at 15°C as compared to 25°C with 3 fold increase as per the RT-PCR data (Figure 11); RPKM based expression analysis also confirmed the same trend (Figure 8).

It was encouraging to note that except for *PAL*, *4-CH*, *COMT*, *3-hydroxy-3-methylglutaryl-CoA synthase*, *HMGR*, *MVK*, *PMK*, *diphosphomevalonate decarboxylase*, the expression of rest of the 11 genes as analyzed by RPKM and RT-PCR was in accordance with each other. The different trend of expression by RPKM and RT-PCR method has been reported by other groups as well [93]. The possibility of opposite results by the two methods could be due to the reasons that genome analyzer provides a holistic picture of all the isoforms of a gene into consideration, whereas the expression by RT-PCR is specific to the isoform of the gene into consideration owing to the use of gene specific primers.

## Conclusions

In this study, transcriptome for leaf tissue of *P. kurrooa *was generated and analyzed for the plants kept at 15°C and 25°C with a total of 74,336 assembled sequences. PE read data along with optimized parameters and suitable multiple assembling and clustering approaches were used to find out non-inflated number of assembled transcript sequences with high coverage and average length. The similarity and overlap based similarity search and assembling might result into inflated number of assembled sequences, many of which could be either from different parts of the same gene or the isoforms. The dissimilar sequence clustering approach used in the present work helped to overcome the above problems to a large extent and reduced over-representation of assembled sequences. GO, KEGG and EC based tools and scripts were used for sequence annotation. Transcriptome data exhibited GC content representative of a dicot genome and also abundance of trinucleotide SSR markers was evident. RPKM based expression analysis by comparing transcriptomes at two temperatures showed major adjustments reflecting changes in major biological processes and metabolic pathways including the pathways associated with picrosides biosynthesis. A number of novel candidate genes involved in picrosides biosynthesis, including *CYPs *and *UGTs *were also identified, which could serve as a source of hitherto uncharacterized genes associated with picrosides biosynthesis. Transcriptome data generated in the present work has immense implications in understanding plant response at two temperatures, marker assisted selection, and metabolic engineering in an economically important, medicinal and endangered plant species *P. kurrooa*.

## Methods

### Plant Material

Plants of *P. kurrooa *were collected from its natural habitat at Rohtang pass (4,000 m altitude, 32°23' N, 77°15' E, India) and maintained at the Institute at Palampur (1,300 m altitude; 32°06' N, 76°33' E, India) as described previously [5]. After three months at Palampur, these were shifted to plant growth chambers (Percival Scientific, USA) maintained at 15°C and 25°C with a 16-h photoperiod. Plants were adequately watered and the sampling was done on day 6 at the two temperatures. Third leaf (position with respect to the top apical leaf designated as first leaf) was harvested for various experiments, frozen in liquid nitrogen and stored at -80°C for further use.

### Extraction and estimation of picrosides

Picrosides were estimated as described previously [94] except that Ultra Performance Liquid Chromatography (UPLC) system consisting of Acquity UPLC (Waters, Millford, USA) equipped with binary solvent manager, sample manager, photodiode array detector (PDA) and a BEH workflow Shield C_18 _(1.7 μm particles, 2.1 × 100 mm) analytical column (Waters Corp., Manchester UK) was used. After extraction, samples were filtered through 0.22 micron filter (Millipore, USA) and injected into the chromatographic system. The mobile phase consisted of formic acid (0.05%) in water and methanol:acetonitrile (1:1) in 70:30 ratio. Isocratic elution was carried out at a flow rate of 0.250 ml min^-1 ^with injection volume of 5 μl. Picrosides were monitored at 270 nm and quantified using picroside I and picroside II as standards (ChromaDex™, USA). Four separate biological replicates were used for each estimation.

### Preparation of cDNA and transcriptome sequencing

Total RNA was extracted as described by Ghawana et al. [95]. Quality and quantity of RNA was determined using a Nanodrop 1000 (NanoDrop Technologies, USA) and a Bioanalyzer Chip RNA7500 series II (Agilent Technologies, USA). Total RNA was used to purify poly (A) mRNA using Oligotex mRNA midi prep kit (QIAGEN, Germany) followed by repurification using mRNA-Seq 8 sample prep kit (Illumina, USA). This was used to prepare a non-directional Illumina RNA Seq library. Quality control and quantification of library was performed with a Bioanalyzer chip DNA 1200 series II (Agilent Technologies, USA). Each library had an average insert size of 200 bp. PE 36 bp sequences were generated on Illumina genome analyzer IIx following manufacturer's instructions.

### *De novo *assembly and sequence clustering

**Figure 12:**
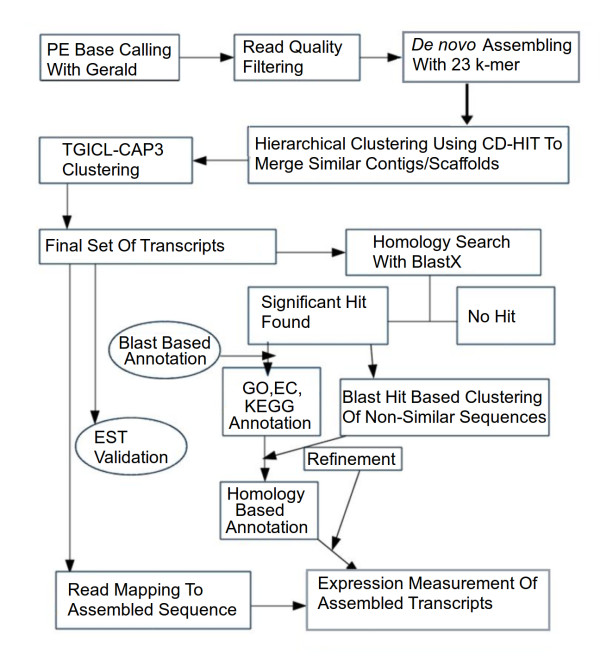
**Workflow of *de novo *transcriptome assembly, annotation and reads per exon kilobase per million (RPKM) based expression analysis**.

The assembled transcript sequences, the filtered read data, transcript grouping for similar genes, are available at http://scbb.ihbt.res.in/Picro_information. Entire computational analysis was carried out on CentOS based 48 cores 2.2 Ghz AMD processors based HPC server with 256 GB random access memory (RAM) as well as Ubuntu Linux based workstations with 8 cores 2.5 Ghz Intel processors with 24 GB RAM. Using CASAVA package GERALD tool, provided by Illumina, PE sequence reads were generated in fastq format. For each lane, PE reads of length 36 were generated, with total 72 bp. Last three base pairs from each read were removed in order to minimize the sequencing error, which is usually higher in the 3' end of reads. An in-house developed tool, filteR, was used to filter out poor quality reads. FilteR was developed using C++ to detect adapter sequence contamination as well as poor read quality. In its back-end it applies the quality scoring scheme provided by Illumina. It also provides an option, Recommender, which allows the user to decide the suitable cut-off to perform read screening by calculating average read quality positionally. This allows selective trimming of the reads instead of discarding the entire read. To attain fast processing and take advantage of multicore processors, concurrency has been introduced in it using openMP. Besides this, it also provides a user friendly GUI implemented using Qt C++ library. FilteR has been made freely available for the community at http://scbb.ihbt.res.in/SCBB_dept/filter.php. K-mer frequency measurement was performed to filter out reads with lower k-mer frequency for default value, which could be a result of sequencing error. *De novo *assembling of high quality reads was performed using SOAPdenovo program which applies de Bruijn graph algorithm and a series of stepwise strategies [15]. The cleaned reads were first split into smaller pieces, the 'k-mers', for assembly in order to produce contigs, using the de Bruijn graph. K-mer size of 23 achieved the best balance between the number of contigs produced, coverage and average sequence length attained. PE option of assembling with distance of 200 bp was applied to assist more effective assembling with information of paired reads. The same information was also used to build the scaffold sequences by merging two contigs into single scaffold sequence, sharing the read pairs. Figure 12 shows the protocol used in *de novo *assembling and transcript analysis of assembled sequences for a given sample. Sequence redundancy was removed by searching similar sequences with minimum similarity cut-off of 95% using CD-HIT-EST [96]. CD-HIT was used for further clustering with 90% similarity cut-off. The algorithms for various clustering programs differed in their approach of clustering and combined use of such clustering tools with different algorithms fetched better results [97]. For the same reason clustering process was supplemented with TGICL-CAP3 clustering [98] based on terminal region matching for at least 40 bp and 90% identity. The resulting singletons and consensus contigs were merged to get the final list of assembled transcripts. A set of script was developed to detect contigs/scaffolds that had no sequence similarity but belonged to same gene's different regions. These were clustered together to represent as a single transcript. The best BLASTX hits for all contigs were looked for common NR database ID for a particular gene/peptide and all associated contigs showing highest similarity to the same sequence but its different regions, were assigned to the same ID group.

### Assembly validation and similarity search for assembled transcripts

In order to assess the reliability of assembly, 500 experimentally validated EST sequences available at dbEST NCBI of *P. kurrooa *were used (Additional file 5). A BLASTN analysis was performed for each reported EST against the set of assembled sequences at E-value threshold of 10^-5^.

For similarity search, the assembled and filtered transcript sequences obtained after hierarchical clustering were scanned against NR protein database [99] through BLASTX with the E-value threshold of 10^-5^.

### Sequence annotation

Assembled transcripts of *P. kurrooa *were blasted against UniProt databases [100] and associated GO [101], KEGG [102] and EC with a cut-off E-value of 10^-1^. It was observed that high stringent cut-off missed out either the right candidate and valid annotations and sometimes no hit was reported. GO terms were assigned for each unigene based on the GO terms annotated to its corresponding homologue in the UniProt database. For every query transcript sequence multiple hits were observed. Using in-house developed scripts, the best hit observed for each given sequence was selected based on highest bitscore and E-value. Majority of GO, EC and KEGG based annotation and statistics was done using Annotation tool, Annot8r [103]. Plant transcription factor database [104] hosts large number of plant specific TFs, their classification, corresponding nucleic acids and protein sequences. In the current study, data for all the 29,474 TFs reported in the database, version 3.0, were downloaded. The assembled transcript sequences were searched against this database using BLASTX with an E-value threshold of 10^-5^. Only the top bit-scoring significant hit for each sequence was considered. GO term enrichment analysis was performed using Bingo tool [105] for hyper-geometric test, with Bonferroni Family-wise error rate correction [106]. This enrichment analysis was performed to evaluate the enrichment of various GO categories for the transcripts having expression level 2 fold or above at 15°C and 25°C.

### Functional domains search for unknown sequences

The assembled sequences which did not return any homologous sequence hit through BLASTX, were converted into six longest ORFs, which were scanned against the functional domain databases like Conserved domain database, using RPS-BLAST [107] using UNIX version of RPS-BLAST [108,109].

### Comparative similarity search for assembled sequences for different environmental conditions

In order to find the common transcripts between sets of assembled sequences for *P. kurrooa *at 15°C and 25°C, the assembled transcript sequences for both the conditions were search against each other using BLASTN with E-value threshold of 10^-5^. Transcripts returning best hits were identified as the common transcripts.

### Read mapping and transcript abundance measurement

Applying the approach adopted by Mortazvi et al. [110] the expression level of each assembled transcript sequence can be measured through RPKM values. RPKM level measurement is a sensitive approach to detect expression level, that measures expression of even poorly expressed transcripts using read count as the fundamental. For RPKM measurement, we first mapped back the filtered reads to various assembled transcripts, estimated total mapped reads, uniquely mapped reads assigned to each assembled transcript, with maximum two mismatches allowed. SeqMap [111] was used for read mapping and rSeq [112] was applied for RPKM based expression measurement. Expression data from both the sample were collected for each of the transcripts. Similar sequences across the samples were searched and their differential expression was measured by calculating the ratio of expression at 15°C and 25°C. Assembled sequences were used as the reference sequence to map back short reads and to measure RPKM for all assembled transcripts as suggested by Mortazavi et al. [110] and Jiang and Wong [111]. Based on the above mentioned dissimilar sequence clustering, having homologous sequences in database, for each such cluster, the longest sequence was considered as the representative sequence for the unique gene it represented. The associated GO terms and Ids were parsed for each of such sequence and their corresponding RPKM values for the two different temperature conditions were calculated along with fold increment.

### GC content analysis and SSRs identification

GC content of the sequences was measured using Emboss GeeCee tool, while sequences were scanned for SSR markers using MISA [113].

### Gene validation and expression analysis

Results of gene expression were validated by RT-PCR. RNA was pretreated with RNase-free DNase I (Invitrogen, USA) to remove any contaminating DNA followed by first strand cDNA synthesis with 1 μg of total RNA using superscript III (Invitrogen, USA) according to the manufacturer's instructions. PCR conditions including primer details for RT-PCR are mentioned in Additional file 16. Cycling conditions were optimized to obtain amplification within the exponential phase. Amplicons were quantified using Alpha DigiDoc gel documentation and image analysis system (Alpha Innotech, USA). Triplicates of each reaction were performed, and *26 s rRNA *was chosen as an internal control for normalization [92].

## Competing interests

The authors declare that they have no competing interests.

## Authors' contributions

PG carried out experiment at 15°C and 25°C, prepared cDNA library for Illumina sequencing, measured picrosides content and performed expression analysis, HRS performed read generation, process of assembling, clustering, homology searching, annotation, CDD search, SSR markers discovery and entire computational analysis. NS performed sequencing run and expression analysis, AK performed sequencing run and cDNA library preparation for Illumina sequencing, VC developed the parallel coded filtering tool, FilteR, performed read filtering and GO functional enrichment analysis with hypergeometric tests. RS conceived, planned, developed and tested the protocols for the entire computational part of this study, performed reads based expression analysis and associated studies, developed the algorithm and tool for dissimilar sequence clustering and supervised the entire computational part of the study. PSA supported the work. SK conceived the study, designed the experiment, associated with wet lab work, results interpretation, analysis and integration of results and coordinated the study. PG, HRS drafted the manuscript. RS and SK drafted and finalized the manuscript. All authors have read and approved the manuscript.

## Supplementary Material

Additional file 1 **Pooled transcriptome data of *P. kurrooa***. Due to large amount, data is divided into Additional file 1 and Additional file 2. Click here for file

Additional file 2 **Pooled transcriptome data of *P. kurrooa***. File contains the remaining data of Additional file 1. Click here for file

Additional file 3 **Dissimilar sequence groupings for assembled transcriptome sequences**. The grouped sequences are dissimilar from each other, and belong to different regions of common genes. This helps reducing the over-representation of total transcripts, usually missed in assembling. Click here for file

Additional file 4 **Top highly represented functional conserved domains found in unknown sequences**. Click here for file

Additional file 5 **Experimentally validated expressed sequence tags (ESTs) of *P. kurrooa *available at dbEST NCBI**. Methods section has details including URL used in the present analysis. Click here for file

Additional file 6 **Guanine-cytosine (GC) content analysis of *P. kurrooa *transcripts**. The average GC content of each transcript was calculated and highest proportion of transcripts belongs to the GC content range of 40-49%. Click here for file

Additional file 7 **Simple sequence repeats (SSRs) identified in transcripts of *P. kurrooa***. Click here for file

Additional file 8 **Comparative plots for top ten highest represented molecular function (A) and biological process (B) categories in *A. thaliana *and *P. kurrooa***. Click here for file

Additional file 9 **Reads per exon kilobase per million (RPKM) based gene expression at 15°C and 25°C in different expression categories ranging from > 10 fold increment to 0.5 fold or lower**. Click here for file

Additional file 10 **Details on all the transcription factor (TF) families analyzed in *P. kurrooa***. Click here for file

Additional file 11 **Top 20 most abundant transcription factor (TF) families analyzed in *P. kurrooa***. Details of all the TFs are mentioned in Additional file 10. Click here for file

Additional file 12 **Reads per exon kilobase per million (RPKM) based expression of transcription factors analysed for *P. kurrooa *transcriptome at 15°C and 25°C**. Click here for file

Additional file 13 **Reads per exon kilobase per million (RPKM) based expression of targeted genes of mevalonate (MVA), 2-C-methyl-D-erythritol 4-phosphate (MEP) and phenylpropanoid (PP) pathway (pathways associated with picrosides biosynthesis) at 15°C and 25°C**. Click here for file

Additional file 14 **Reads per exon kilobase per million (RPKM) based expression of annotated *cytochrome P450s *(*CYPs*) in *P. kurrooa *transcriptome**. Click here for file

Additional file 15 **Reads per exon kilobase per million (RPKM) based expression of annotated *glycosyltransferases *in *P. kurrooa *transcriptome**. Click here for file

Additional file 16 **Oligonucleotide sequences and polymerase chain reaction (PCR) conditions used in reverse transcriptase (RT-PCR) based expression analysis**. Click here for file
