# Supplementary figures and images for: De novo sequencing and characterization of Picrorhiza kurrooa transcriptome at two temperatures showed major transcriptome adjustments
Source: BMC Genomics. 2012 Mar 31;13:126. doi: 10.1186/1471-2164-13-126 (PMC3378455; doi:10.1186/1471-2164-13-126)

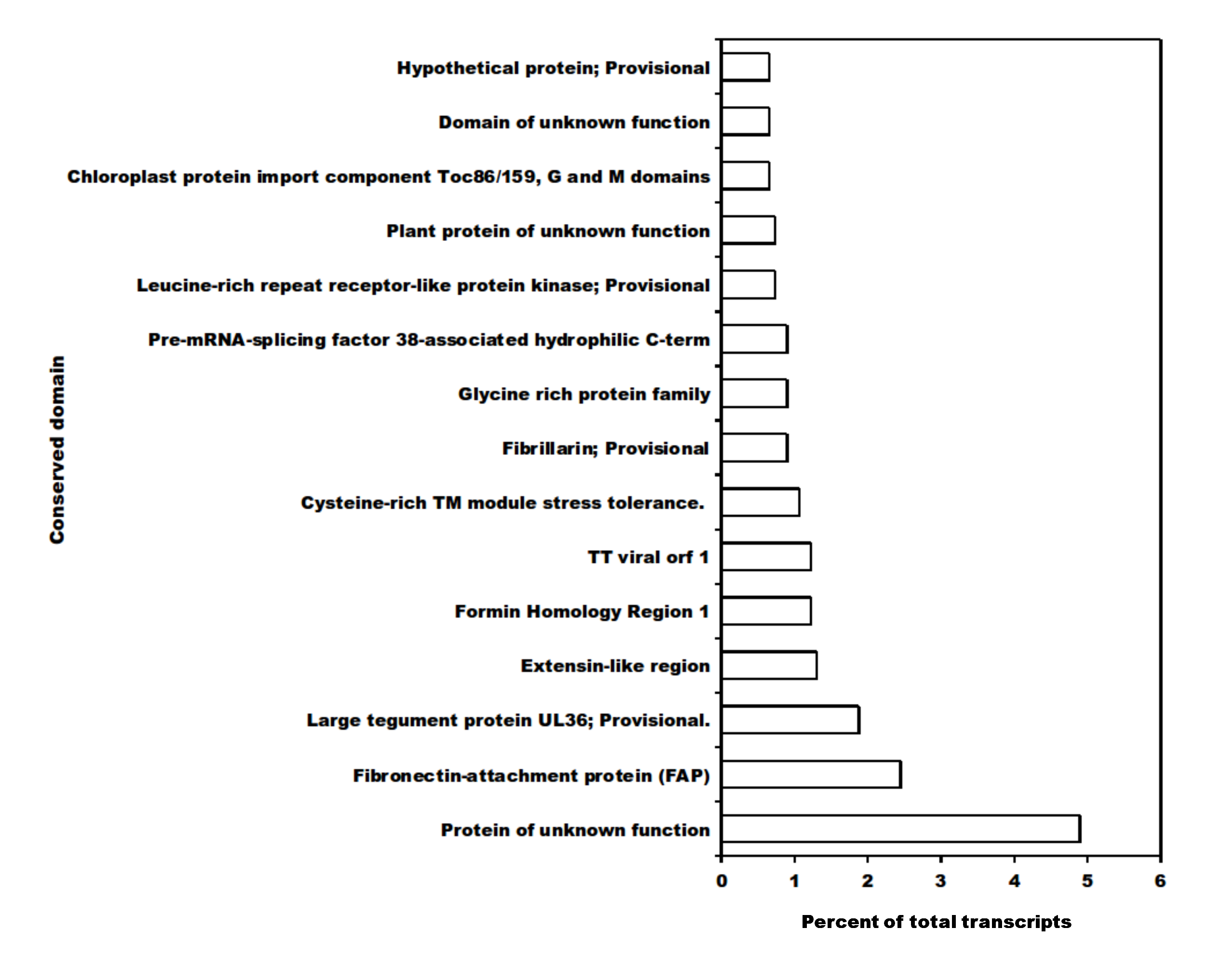

Supplement: Additional file 4 — Top highly represented functional conserved domains found in unknown sequences. [file 1471-2164-13-126-S4.TIFF]

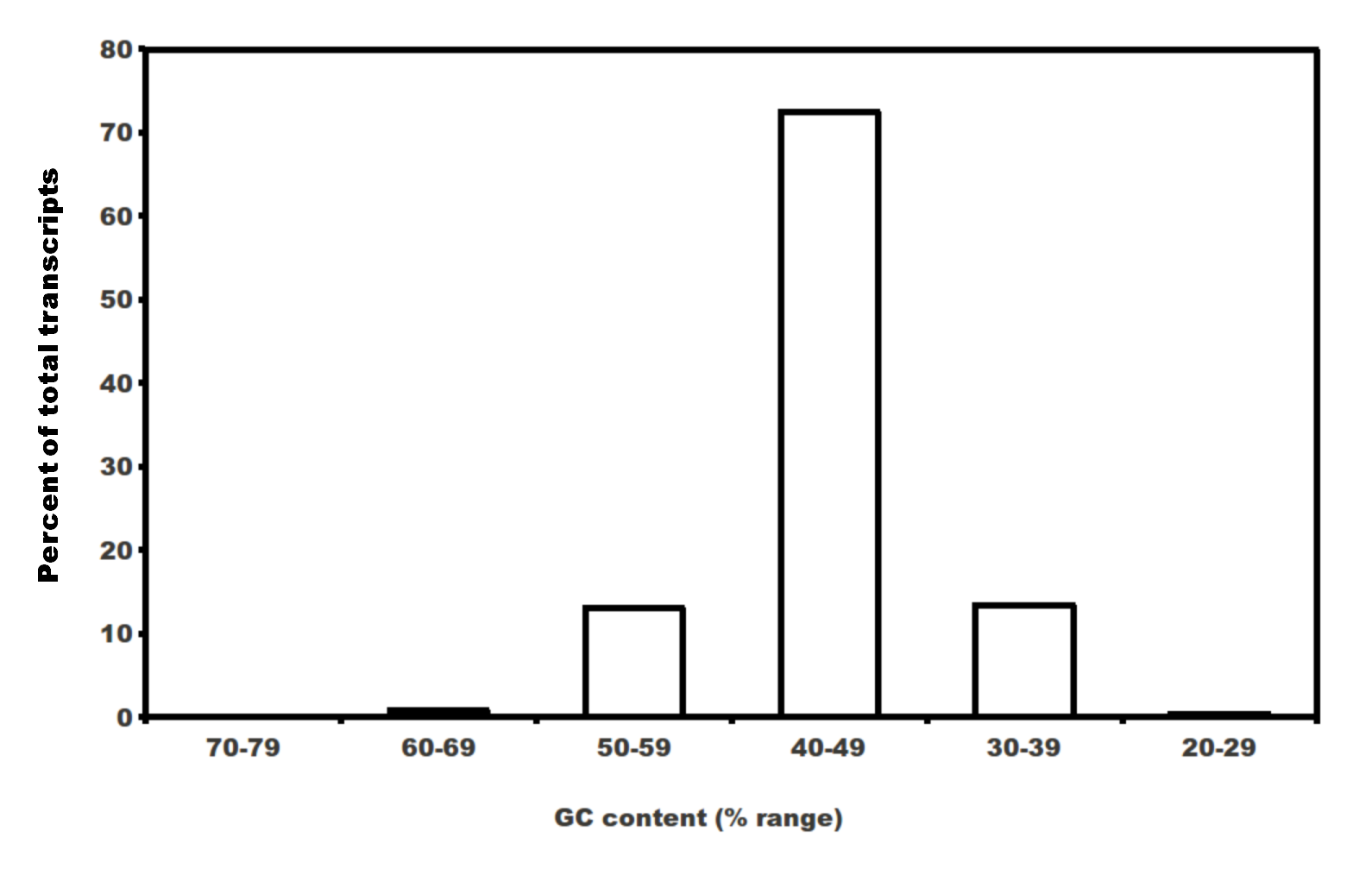

Supplement: Additional file 6 — Guanine-cytosine (GC) content analysis of P. kurrooa transcripts. The average GC content of each transcript was calculated and highest proportion of transcripts belongs to the GC content range of 40-49%. [file 1471-2164-13-126-S6.TIFF]

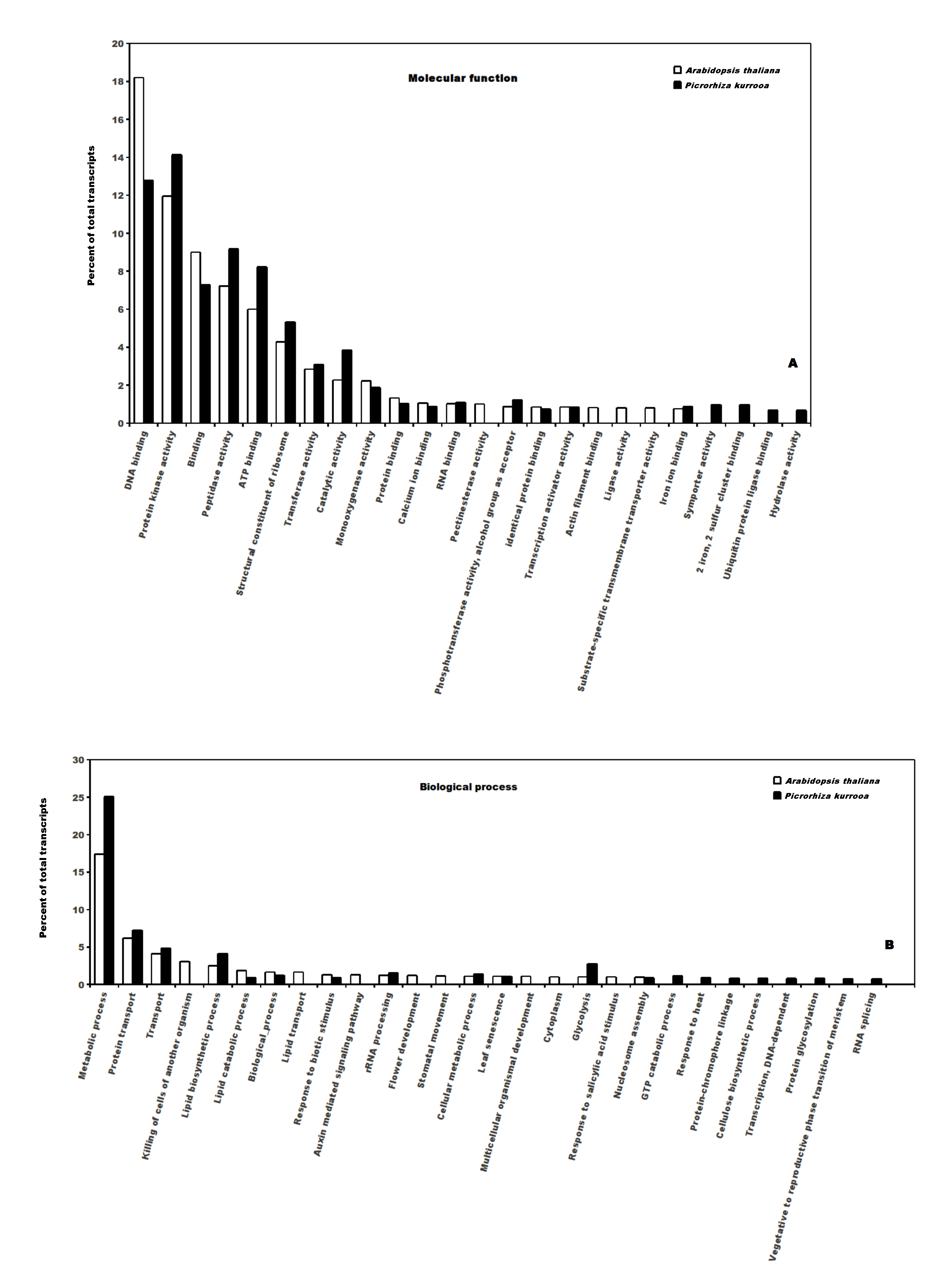

Supplement: Additional file 8 — Comparative plots for top ten highest represented molecular function (A) and biological process (B) categories in A. thaliana and P. kurrooa. [file 1471-2164-13-126-S8.TIFF]

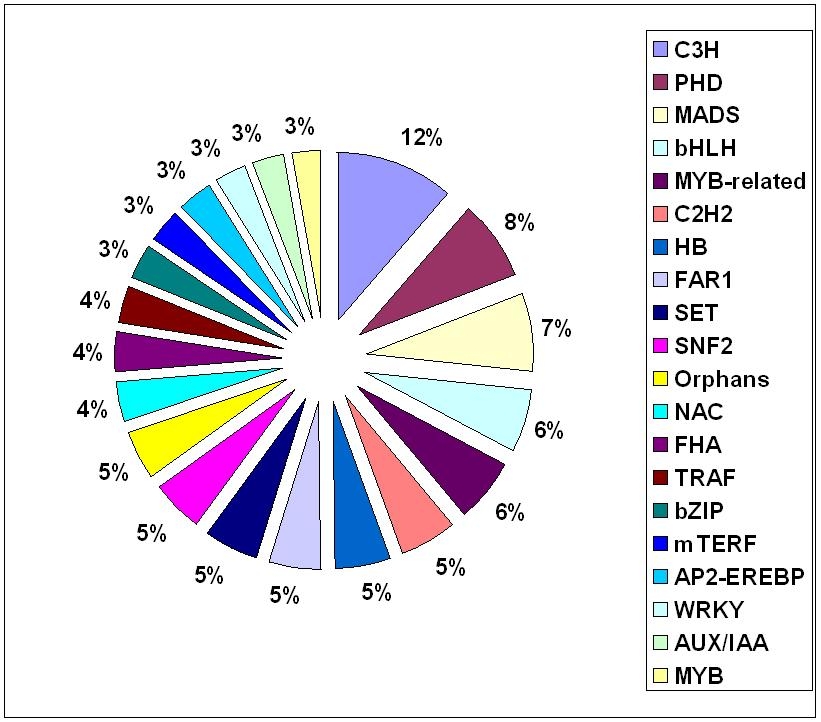

Supplement: Additional file 11 — Top 20 most abundant transcription factor (TF) families analyzed in P. kurrooa. Details of all the TFs are mentioned in Additional file 10. [file 1471-2164-13-126-S11.JPEG]
