# Supplementary material for: De novo sequencing and characterization of Picrorhiza kurrooa transcriptome at two temperatures showed major transcriptome adjustments
Source: BMC Genomics. 2012 Mar 31;13:126. doi: 10.1186/1471-2164-13-126 (PMC3378455; doi:10.1186/1471-2164-13-126)
Supplement: Additional file 7 — Simple sequence repeats (SSRs) identified in transcripts of P. kurrooa. [file 1471-2164-13-126-S7.DOC]

Simple sequence repeats **(**SSRs) identified in transcripts of *P. kurrooa*

| **SSR mining** | |
| --- | --- |
| Total number of sequences examined: | 72,220 |
| Total size of examined sequences (bp): | 32,400,167 |
| Total number of identified SSRs: | 1,562 |
| Number of SSR containing sequences: | 1,512 (0.02%) |
| Number of sequences containing more than one SSR: | 48 |
| Number of SSRs present in compound formation: | 17 |
|  |  |
| **Distribution of SSRs in different repeat types** | |
| **Unit size** | **Number of SSRs** |
| Mononucleotide | 533 (35.25%) |
| Dinucleotide | 322 (21.29%) |
| Trinucleotide | 690 (45.63%) |
| Tetranucleotide | 14 (0.009%) |
| Pentanucleotide | 3 (0.002%) |
