# Supplementary material for: De novo sequencing and characterization of Picrorhiza kurrooa transcriptome at two temperatures showed major transcriptome adjustments
Source: BMC Genomics. 2012 Mar 31;13:126. doi: 10.1186/1471-2164-13-126 (PMC3378455; doi:10.1186/1471-2164-13-126)
Supplement: Additional file 13 — Reads per exon kilobase per million (RPKM) based expression of targeted genes of mevalonate (MVA), 2-C-methyl-D-erythritol 4-phosphate (MEP) and phenylpropanoid (PP) pathway (pathways associated with picrosides biosynthesis) at 15°C and 25°C. [file 1471-2164-13-126-S13.DOC]

| **S.No.** | **Gene name** | **Transcript Length (bp)** | **Transcript ID** | **Expression at 15 °C** | **Expression at 25 °C** | **Fold expression at 15 °C** |
| --- | --- | --- | --- | --- | --- | --- |
| 1 | 1-deoxy-D-xylulose 5-phosphate synthase | 1573 | 54959_scaffold30219 149.6 | 143.123 | 68.203 | 2.098 |
| 2 | 1-deoxy-D-xylylose 5-phosphate reductoisomerase | 598 | 35022_scaffold12179 68.9 | 157.56 | 69.867 | 2.255 |
| 3 | 2-C-methyl-D-erythritol 4-phosphate cytidylyltransferase | 1036 | 68214_scaffold4663 148.1 | 28.73 | 30.649 | 0.937 |
| 4 | 4-(cytidine 5'-diphospho)-2-C-methyl-D-erythritol kinase | 287 | 30088_C811605 211.0 | 167.50 | 96.176 | 1.741 |
| 5 | 2-C-methyl-D-erythritol 2,4-cyclodiphosphate synthase | 302 | 30412_C815021 196.0 | 222.12 | 102.057 | 2.176 |
| 6 | 4-hydroxy-3-methylbut-2-enyl diphosphate synthase | 1407 | 45781_scaffold21922 107.6 | 54.69 | 42.937 | 1.273 |
| 7 | 4-hydroxy-3-methylbut-2-enyl diphosphate reductase | 815 | 72271_scaffold8343 116.3 | 183.75 | 96.245 | 1.90 |
| 8 | acetyl-CoA acetyltransferase | 1552 | 67053_scaffold41133 75.3 | 67.75 | 73.212 | 0.925 |
| 9 | 3-hydroxy-3-methylglutaryl-CoA synthase | 1530 | 49024_scaffold24855 194.0 | 28.81 | 56.926 | 0.506 |
| 10 | 3-hydroxy-3-methylglutaryl-CoA reductase | 1691 | 44484_scaffold20750 78.1 | 10.56 | 17.821 | 0.592 |
| 11 | mevalonate kinase | 1245 | 65322_scaffold39573 59.5 | 9.06 | 17.212 | 0.526 |
| 12 | 5-phosphomevalonate kinase | 1157 | 58478_scaffold33394 60.5 | 9.77 | 17.007 | 0.574 |
| 13 | 5-diphosphomevalonate decarboxylase | 340 | 47868_scaffold23811 81.1 | 19.14 | 37.601 | 0.508 |
| 14 | isopentenyl diphosphate-dimethylallyl diphosphate isomerase | 641 | 63658_scaffold38072 202.9 | 100.64 | 106.854 | 0.941 |
| 15 | geranyl diphosphate synthase | 1573 | 42915_scaffold19333 140.2 | 67.69 | 31.3122 | 2.161 |
| 16 | monoterpene synthase/terpene synthase | 1485 | 39328_scaffold16079 6.6 | 78.76 | 110.933 | 0.710 |
| 17 | monoterpene cyclase/terpene cyclase | 1380 | 63964_scaffold38349 47.5 | 98.52 | 47.248 | 2.085 |
| 18 | phenylalanine ammonium-lyase | 547 | 34495_scaffold117 109.4 | 20.04 | 327.448 | 0.061 |
| 19 | 4 coumarate CoA: ligase | 1214 | 32553_C838979 250.0 | 46.23 | 77.945 | 0.593 |
| 20 | cinnamic acid 4-hydroxylase | 490 | 50196_scaffold25916 85.1 | 166.54 | 226.631 | 0.734 |
| 21 | coumarate 3-hydroxylase | 1911 | 61139_scaffold35799 105.9 | 13.86 | 36.484 | 0.379 |
| 22 | caffeoyl-CoA 3-O-methyltransferase | 768 | 32406_C837369 144.0 | 35.53 | 57.029 | 0.623 |
