# Supplementary material for: De novo sequencing and characterization of Picrorhiza kurrooa transcriptome at two temperatures showed major transcriptome adjustments
Source: BMC Genomics. 2012 Mar 31;13:126. doi: 10.1186/1471-2164-13-126 (PMC3378455; doi:10.1186/1471-2164-13-126)
Supplement: Additional file 16 — Oligonucleotide sequences and polymerase chain reaction (PCR) conditions used in reverse transcriptase (RT-PCR) based expression analysis. [file 1471-2164-13-126-S16.DOC]

**Primers for expression studies**

| **Name** | **Sequence (5′-3′)** | **PCR Conditions** |
| --- | --- | --- |
| *DXS* F | CGTCGATTGTAGCTGTAGGC | Initial denaturation at 94 °C for 3 min, followed by 30 cycles of 94°C, 30 s; 59 °C, 40 s; 72 °C, 1 min. Final extension at 72 °C for 7 min |
| *DXS* R | TATCGGTGATTGGAGATGGA |
| *DXR* F | CTTTTCGGGGCAGAATATGA | Initial denaturation at 94 °C for 3 min, followed by 30 cycles of 94 °C, 30 s; 59 °C, 40 s; 72 °C, 1 min. Final extension at 72 °C for 7 min |
| *DXR* R | GCAGCATAAGCCAAATCCAT |
| *MCT* F | AGGGAAGGACTTGAAGTAACAGA | Initial denaturation at 94 °C for 3 min, followed by 28 cycles of 94 °C, 30 s; 57 °C, 40 s; 72 °C, 1 min. Final extension at 72 °C for 7 min |
| *MCT* R | GTGGAGGAACAATGCTCAGA |
| *CMK* F | AGAATGGTCTGGCGAGATTG | Initial denaturation at 94 °C for 3 min, followed by 30 cycles of 94 °C, 30 s; 57 °C, 40 s; 72 °C, 1 min. Final extension at 72 °C for 7 min |
| *CMK* R | AGGACTTCAAAAGCCGGAAT |
| *MDS* F | CCAAGTAGCACGCACAAGTT | Initial denaturation at 94 °C for 3 min, followed by 28 cycles of 94 °C, 30 s; 58 °C, 40 s; 72 °C, 1 min. Final extension at 72 °C for 7 min |
| *MDS* R | GGGAAATTTAGACGCCACAT |
| *HDS* F | GCTGCAACAGTTGAACAGGTA | Initial denaturation at 94 °C for 3 min, followed by 28 cycles of 94 °C, 30 s; 58 °C, 40 s; 72 °C, 1 min. Final extension at 72 °C for 7 min |
| *HDS* R | TGAATCTGCTTCTTTCCTTCC |
| *HDR* F | GATTGGGAAATTGGCTGAGA | Initial denaturation at 94 °C for 3 min, followed by 29 cycles of 94 °C, 30 s; 57 °C, 40 s; 72 °C, 1 min. Final extension at 72 °C for 7 min |
| *HDR* R | CCAACTCACCATGCATCAAC |
| *AACT* F | GAAGAGGAGGACCATCCACA | Initial denaturation at 94 °C for 3 min, followed by 28 cycles of 94 °C, 30 s; 58 °C, 40 s; 72 °C, 1 min. Final extension at 72 °C for 7 min |
| *AACT* R | GCCGGACCAGTGGTAAATAA |
| *HMGR* F | ACTGATTTACCACGCCCTTC | Initial denaturation at 94 °C for 3 min, followed by 35 cycles of 94 °C, 30 s; 57 °C, 40 s; 72 °C, 1 min. Final extension at 72 °C for 7 min |
| *HMGR* R | TGGTTTCCAAAGGTGTTCAG |
| *HMGS* F | AAGTAGTCAGCATCAGTAACTGA | Initial denaturation at 94 °C for 3 min, followed by 32 cycles of 94 °C, 30 s; 58 °C, 40 s; 72 °C, 1 min. Final extension at 72 °C for 7 min |
| *HMGS* R | AATATCCAGTAGTTGATGGCAAG |
| *MVK* F | TCGCGCTCTCTGCTGCCCTA | Initial denaturation at 94 °C for 3 min, followed by 30 cycles of 94 °C, 30 s; 56 °C, 40 s; 72 °C, 1 min. Final extension at 72 °C for 7 min |
| *MVK* R | CCATTTATTCACCAATTCCAGCTC |
| *PMK* F | CTGGGATCATGAGAGGACTA | Initial denaturation at 94 °C for 3 min, followed by 30 cycles of 94 °C, 30 s; 56 °C, 40 s; 72 °C, 1 min. Final extension at 72 °C for 7 min |
| *PMK* R | GCTGACAACTTCTTCCATGT |
| *PMD* F | ATTAGTGTAACTCTGGATCCTGA | Initial denaturation at 94 °C for 3 min, followed by 30 cycles of 94 °C, 30 s; 58 °C, 40 s; 72 °C, 1 min. Final extension at 72 °C for 7 min |
| *PMD* R | CAGTAGGGAAATCAATGGTAG |
| *IPP* F | GCTGCTCAGAGGAAGCTGTT | Initial denaturation at 94 °C for 3 min, followed by 28 cycles of 94 °C, 30 s; 56 °C, 40 s; 72 °C, 1 min. Final extension at 72 °C for 7 min |
| *IPP* R | GTTTCAAACCCTCCTCACCA |
| *GPS* F | TTGACGAAAGGGGTTTGTTC | Initial denaturation at 94 °C for 3 min, followed by 28 cycles of 94 °C, 30 s; 58 °C, 40 s; 72 °C, 1 min. Final extension at 72 °C for 7 min |
| *GPS* R | GCCCAATACACCTAGCGAAA |
| *MTS* F | CTGATGCAATCCAAAGATGG | Initial denaturation at 94 °C for 3 min, followed by 30 cycles of 94 °C, 30 s; 58 °C, 40 s; 72 °C, 1 min. Final extension at 72 °C for 7 min |
| *MTS* R | ACTTCGCCTCCACCATAAAG |
| *4CH* F | CAACTGAGAAGGCAGGTCAA | Initial denaturation at 94 °C for 3 min, followed by 30 cycles of 94 °C, 30 s; 58 °C, 40 s; 72 °C, 1 min. Final extension at 72 °C for 7 min |
| *4CH* R | ATAAACCCACAAGGCCAGTC |
| *PAL* F | ACTAAACAAGGCGGTGCTCT | Initial denaturation at 94 °C for 3 min, followed by 30 cycles of 94 °C, 30 s; 58 °C, 40 s; 72 °C, 1 min. Final extension at 72 °C for 7 min |
| *PAL* R | GGTGATGGCTTCCAAGATTT |
| *COMT* F | TACATCGGCTACTCACTTCTG | Initial denaturation at 94 °C for 3 min, followed by 33 cycles of 94 °C, 30 s; 57 °C, 40 s; 72 °C, 1 min. Final extension at 72 °C for 7 min |
| *COMT* R | AGTCGAATGTTCCTTCTTCTC |
| *26S* F | CACAATGATAGGAAGAGCCGAC | Initial denaturation at 94 °C for 3 min, followed by 25 cycles of 94 °C, 30 s; 55 °C, 40 s; 72 °C, 1 min. Final extension at 72 °C for 7 min |
| *26S* R | CAAGGGAACGGGCTTGGCAGAATC |

Primers name with “F”, “R” represent forward primers, reverse primers respectively.

Enzymes of MEP pathway are as follows: 1-deoxy-d-xylulose 5-phosphate synthase (DXS), 1-deoxy-d-xylylose 5-phosphate reductoisomerase (DXR), 2-C-methyl-d-erythritol 4-phosphate cytidylyltransferase (MCT), 4-(Cytidine 5′-diphospho)-2-C-methyl-d-erythritol kinase (CMK), 2-C-methyl-d-erythritol 2,4-cyclodiphosphate synthase (MDS), 4-hydroxy-3-methylbut-2-enyl diphosphate synthase (HDS), 4-hydroxy-3-methylbut-2-enyl diphosphate reductase (HDR). Enzymes of MVA pathway are acetoacetyl CoA thiolase (AACT), 3-hydroxy-3-methylglutaryl coenzyme A synthase (HMGS), 3-hydroxy-3-methylglutaryl coenzyme A reductase (HMGR), mevalonate kinase (MVK), phosphomevalonate kinase (PMK), mevalonate-5-pyrophosphate decarboxylase (PMD). isopentenyl pyrophosphate isomerase (IPP isomerase) catalyzes the isomerisation of DMAPP to IPP whereas conversion of IPP to GPP is catalyzed by geranyldiphosphate synthase (GPS). Enzymes of PP pathway are phenylalanine ammonia-lyase (PAL), cinnamate 4-hydroxylase (4 CH), caffeoyl coenzyme A O-methyltransferase
